# Printed Lateral p–n Junction for Thermoelectric Generation

**DOI:** 10.1002/smsc.202400257

**Published:** 2024-08-13

**Authors:** Md Mofasser Mallick, Leonard Franke, Mohamed Hussein, Andres Georg Rösch, Zhongmin Long, Yolita Maria Eggeler, Uli Lemmer

**Affiliations:** ^1^ Light Technology Institute Karlsruhe Institute of Technology (KIT) 76131 Karlsruhe Germany; ^2^ Institute of Microstructure Technology Karlsruhe Institute of Technology (KIT) 76344 Eggenstein‐Leopoldshafen Germany; ^3^ Department of Physics Faculty of Science Ain Shams University Cairo 11566 Egypt; ^4^ Laboratory for Electron Microscopy Karlsruhe Institute of Technology (KIT) 76131 Karlsruhe Germany

**Keywords:** COMSOL, p–n junctions, printed thermoelectrics, Seebeck effects, thermoelectric generators

## Abstract

Printed thermoelectric generators (TEGs) show promising potential for converting waste heat into useful electricity at a low cost but fall short of exhibiting a conversion efficiency anticipated from materials’ properties. The output power of conventionally printed TEGs in the “π‐type” geometry suffers due to low thermal voltage and low current because of high thermal and electrical contact resistance, respectively. Herein, a type of printed p–n junction TEGs (PN‐TEGs) as a possible remedy is explored. Two printed PN‐TEGs with different thicknesses are fabricated using printed p‐type Bi_0.5_Sb_1.5_Te_3_ and n‐type Bi_2_Te_2.7_Se_0.3_ materials. The PN‐TEGs show a promising way to minimize the influence of thermal and electrical resistance in printed TEGs. In the experimental and simulation results, the significant impact of PN‐TEGs’ dimensions on their power outputs is revealed. Also, a conventional “π‐type” printed TEG is fabricated and its performance is studied. The optimized PN‐TEG with a single thermocouple yields ≈14 times higher power output density of 5.3 μW cm^−2^ at a Δ*T* of 25 K compared to “π‐type” printed TEGs.

## Graphical abstract

In this study, printed p–n junction thermoelectric generators (PN‐TEGs) are reported as a possible remedy for the high thermal resistance of conventional TEGs. The printed PN‐TEGs, fabricated with printed p‐type Bi_0.5_Sb_1.5_Te_3_ and n‐type Bi_2_Te_2.7_Se_0.3_ materials, yield ˜14 times higher power output density of 5.3 μW cm^−2^ at a Δ*T* of 25 K compared to conventional “π‐type” printed TEGs.

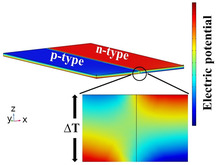

## 1 Introduction

**Figure 1:**
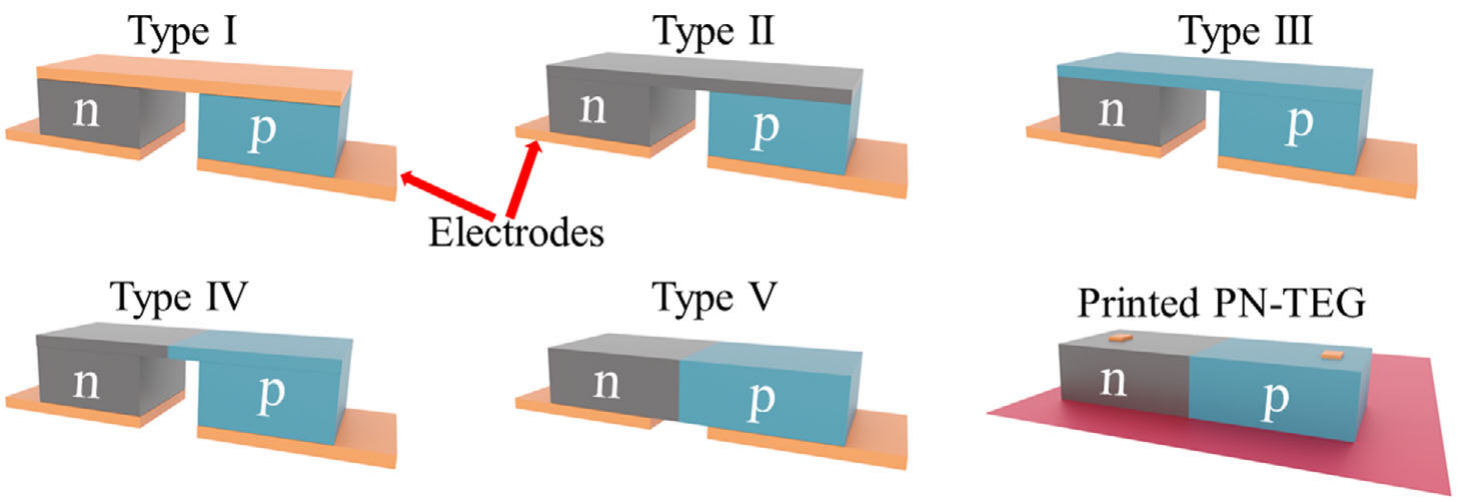
Schematic of different types of TEGs: type I (conventional), type II (n–n–p structure), type III (n–p–p structure), type IV (inverted L shape n–p structure), and type V (p–n structure).

Thermoelectric generators (TEGs) have gained a large interest in both academia and industry due to their ability to convert waste heat into electricity.^[^ ^1^, ^2^, ^3^ ^]^ Further applications of TEGs are in the field of thermal sensing and energy harvesting for autonomous internet of things (IoT) devices.^[^ ^4^, ^5^ ^]^ The first step to fabricate an efficient TEG is to develop a pair of high‐performance n‐ and p‐type thermoelectric (TE) materials. The next step is the optimization of the device using the developed materials.^[^ ^6^ ^]^ The development of a high‐efficiency TE material involves optimization of its charge and phonon transport.^[^ ^7^ ^]^ TE transport properties are considered one of the most interesting phenomena, intertwining with a multitude of fundamental characteristics in solid‐state materials. These include the density of states, bandgap, the effective mass of charge carriers, lattice vibrations, and the intricate realm of phonon scattering in complex materials.^[^ ^8^ ^]^ Altogether the quality of TE materials used to fabricate TEGs is defined by the figure of merit, $zT=S^{2}\sigma T/\kappa$, where *σ, S*, and *κ* are electrical conductivity, the Seebeck coefficient, and the thermal conductivity of the materials.^[^ ^9^ ^]^ Apart from developing a pair of high‐performance n‐ and p‐type TE materials, the fabrication of TEGs with conversion efficiencies in accordance with the TE materials’ properties is of utmost importance.^[^ ^10^ ^]^ Despite decades of thorough research, TEGs have yet to realize the longstanding commitment of widespread applicability due to several fabrication challenges.^[^ ^11^ ^]^ TEG device optimization also requires suitable dimensions and engineering of the device architectures.^[^ ^12^ ^]^ The following well‐known bulk device structures have been studied: 1) type I (conventional TEG); 2) type II; 3) type III; 4) type IV; and 5) type V (c.f. **Figure** **1**).^[^ ^13^ ^]^

However, type I has been predominantly used for printed TEG applications. The TE research community is facing setbacks in fabricating both the bulk and printed TE devices with an efficiency anticipated from materials’ *zT* due to two major issues: 1) lack of electrical and thermal impedance matching; and 2) high electrical and thermal resistance of the devices. The thermal and electrical impedance matching issues can be addressed by selecting suitable dimensions and structures of the TE devices with the help of modeling and simulation.^[^ ^14^ ^]^ However, the thermal resistance of a TEG due to electrical contact layers and insulating substrate materials lowers the thermal voltage significantly. The other challenging part is achieving low electrical contact resistance of TEGs.^[^ ^15^ ^]^ It mainly arises due to atomic diffusion and chemical reactions between the electrodes and the TE materials.^[^ ^16^ ^]^ One way to minimize the contact resistance is by using a diffusion barrier layer between the electrodes and the TE legs.^[^ ^17^ ^]^ Another way is to use low‐diffusive metals like gold as electrodes. When it comes to printed TEGs, however, it becomes more complicated due to the wet printing process of TE materials.^[^ ^18^, ^19^ ^]^ The chemical reactions between the electrodes and the printed TE materials are found to be even more substantial. Therefore, it is imperative to reduce the thermal and electrical contact resistance. If both contact resistances were negligible, the conventional type‐I TE device would exhibit predicted efficiency according to the materials’ properties. In the case of high contact resistances, other types of device architectures might be a better choice.

In an attempt to address this problem, we have employed a type‐V structure to fabricate a printed p–n junction TEG, PN‐TEG. We have used previously developed printed Sb–Bi–Te‐based p‐ and n‐type materials for the PN‐TEG by printing them next to each other on an anodized aluminum substrate (c.f. Figure 1).^[^ ^20^ ^]^ The p and n legs overlapped by 0.1–0.2 mm, which was subsequently flattened by polishing. Although the internal electrical resistance is higher, the thermal contact resistance, which lowers the thermal voltage,^[^ ^21^ ^]^ is found to be minimized in PN‐TEGs compared to printed type‐I (π) TEGs. We have studied the device performance of the two PN‐TEGs with thicknesses 60 and 80 μm for different electrode distances. The power output of the PN‐TEG increases with increasing thickness (*t*) and decreasing electrode distance (*L*). We also demonstrated the comparison between the device performances of a printed type‐V PN‐TEG and a printed type‐1 (π) TEG based on the same materials. It is observed that the power output density of 5.3 μW cm^−2^ for printed PN‐TEG is 14 times higher than for the printed type‐I TEG.

## 2 Experimental Results

**Figure 2:**
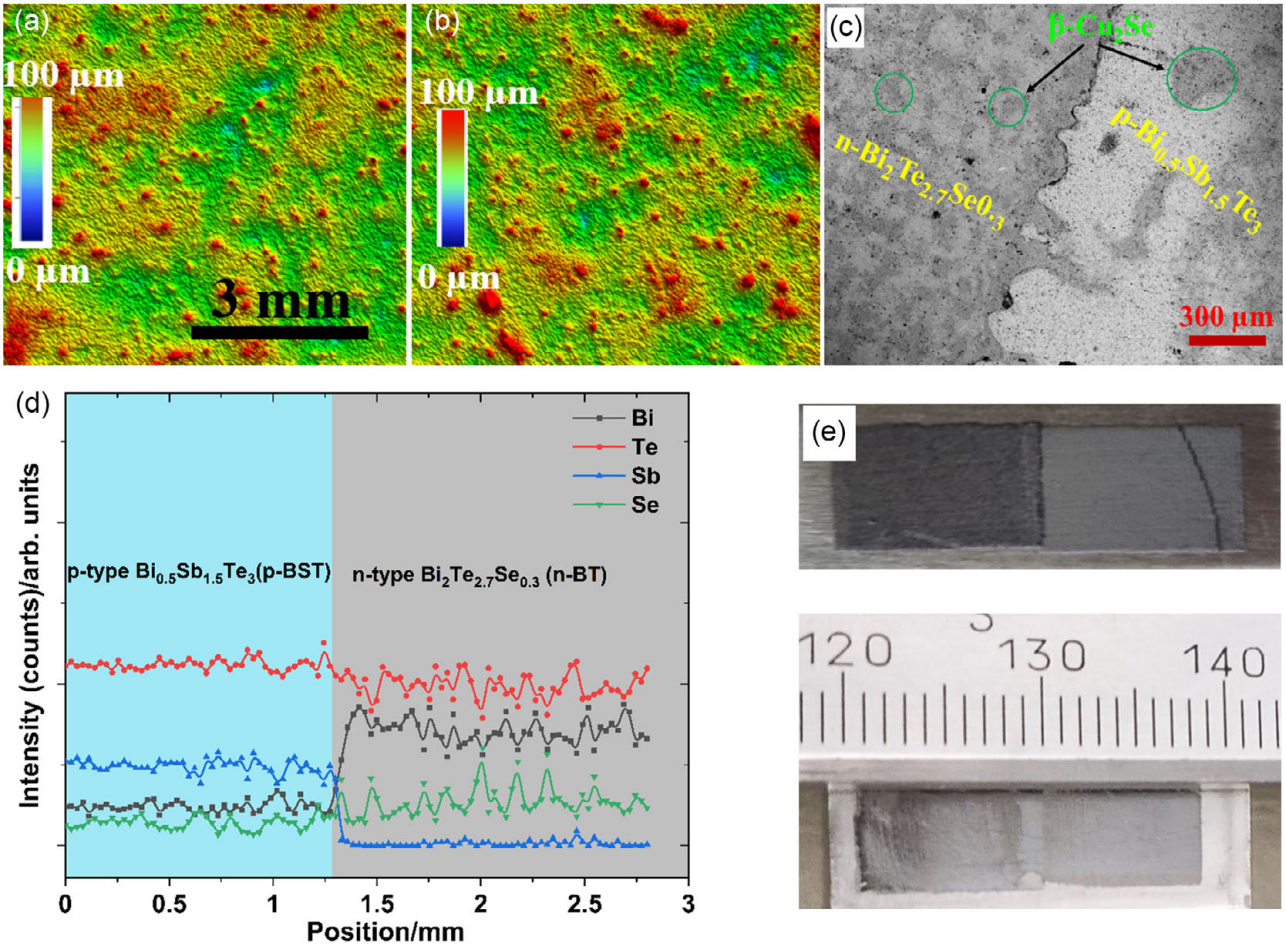
a) Morphology of the printed p‐BST film and b) printed n‐BT film using WLI 3D microscope after sintering. c) Scanning electron microscopic (SEM) image of the interface of p‐BST and n‐BT printed film. d) The arrow indicates the position and direction of the energy‐dispersive X‐ray spectroscopy line scan from the p‐BST film to the n‐BT film. e) The printed p–n junction TE element.

The printed TEGs comprise p‐type Bi_0.5_Sb_1.5_Te_3_ (p‐BST) and n‐type Bi_2_Te_2.7_Se_0.3_ (n‐BT) films which were printed using microparticle inks adjacently with a slight overlap forming a p–n junction using two screens (see Experimental Section). The thickness and morphological properties of the printed p‐BST and n‐BT films were analyzed using a white light interferometer (WLI) after sintering. The surfaces of both the p‐BST and n‐BT printed films are found to be relatively rough at the microscopic scale, which have been polished to study the microstructural and morphological properties of the films using a scanning electron microscope (SEM, see **Figure** **2**a,b). The microstructural properties of a printed p–n junction TE element were analyzed using SEM. Figure 2c,d shows the microstructures of the printed p–n junction TE element at the interface between p‐BST and n‐BT.

It indicates the presence of two distinct phases in both printed p‐ and n‐type films. The spots inside green circles corresponding to β‐Cu_2−δ_Se phase formed through the sintering process.^[^ ^22^ ^]^ β‐Cu_2−δ_Se acts as an inorganic binder (IB) in our composite film. Detailed discussion on β‐Cu_2−δ_Se phase formation and transport properties of the p‐ and n‐type materials has been reported previously.^[^ ^23^ ^]^ A line scan across the interface between the p‐BST and n‐BT printed film has been carried out to identify the change in elemental composition (see Figure 2d). It is found that the at% of Sb decreases, and the at% of the Bi increases drastically at the interface between p‐BST and n‐BT. The presence of the β‐Cu_2−δ_Se phase is also observed at some parts of the interface indicating the diffusion of the β‐Cu_2−δ_Se phase across the interface into the p‐BST and n‐BT region.

**Figure 3:**
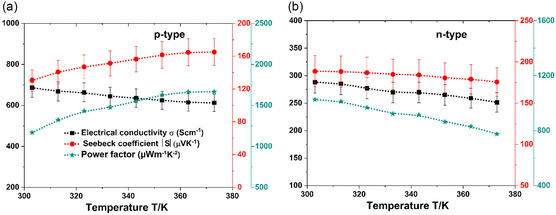
Variation of TE parameters *σ*, *S*, and power factor *S* ^2^ *σ* with temperature for a) printed p‐BST and b) n‐BT films.

**Figure 4:**
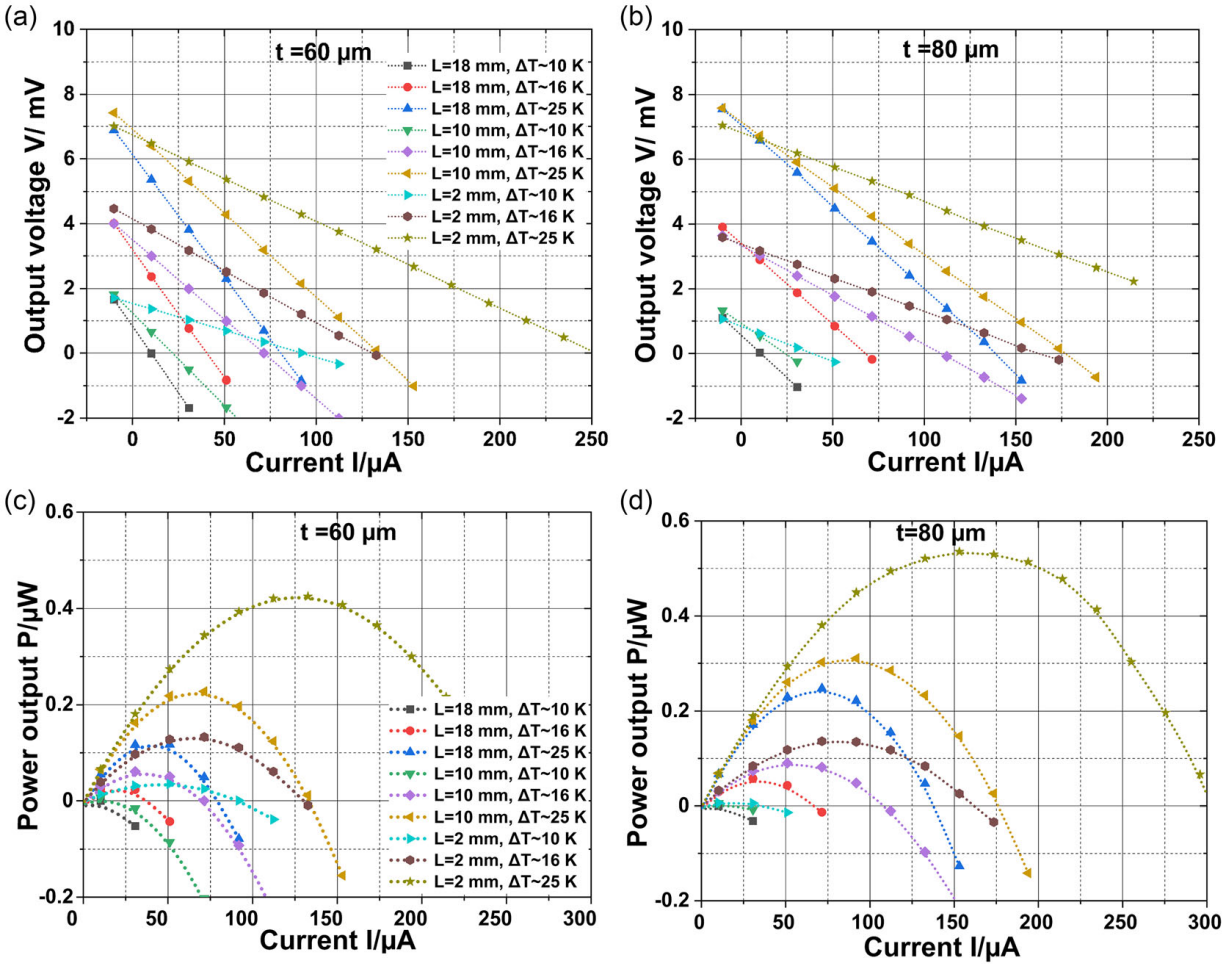
a,b) Variation of output voltages and c,d) power output with the current of the PN‐TEG for two different thicknesses 60 and 80 μm.

**Figure 5:**
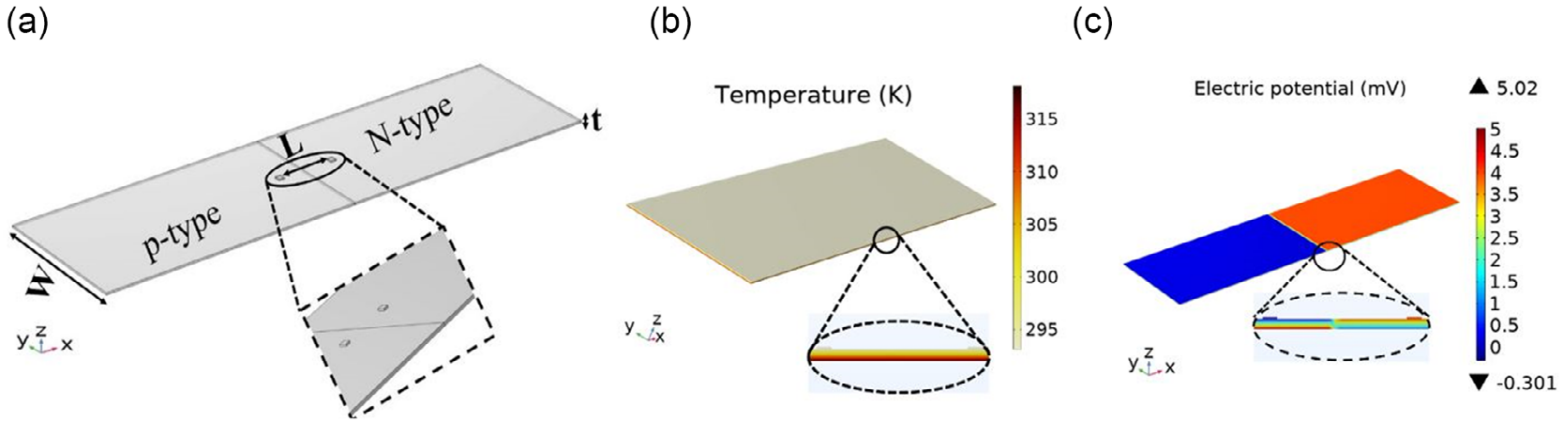
The proposed PN‐TEG: a) schematic diagram, b) temperature distribution, and c) electrostatic potential at Δ*Τ*  = 25 K.

The development of printed p‐BST and n‐BT films and the optimization of their TE properties have been described before.^[^ ^22^ ^]^ The printed p‐BST film containing 5 wt% IB and the printed n‐BT film containing 10 wt% IB are found to be the optimal composition. Both the p‐ and n‐type printed films show a negative temperature coefficient for the conductivity. The p‐BST film exhibits higher *σ* and lower *S* in the complete temperature range. The power factor (*S* ^2^ *σ*) is found to be more than 1000 μW m^−1^ K^−2^ for both the p‐BST and n‐BT printed TE materials (c.f. **Figure** **3**), which are among the highest reported values for pressure‐treatment‐free printed TE materials.^[^ ^24^ ^]^ Hence, the p‐BST and n‐BT printed materials were employed to fabricate printed PN‐TEGs. Here, we have fabricated and carried out a performance analysis of two distinct type‐V p–n junction TE elements, namely PN‐TEG 60 and PN‐TEG 80. Thicknesses of the PN‐TEGs are 60 and 80 μm, respectively, and a uniform width of 5 mm. The fabrication process involved printing n‐BT and p‐BST films side by side using a screen printer, resulting in the creation of p–n junctions. To evaluate the performance of the PN‐TEG 60 and PN‐TEG 80, we employed the maximum power point tracking method, systematically varying the temperature difference (Δ*T*) between the hot side (substrate) and cold side (electrodes) of the PN‐TEGs from 10 to 25 K as shown in Supporting Information (c.f. Figure S1, Supporting Information). We have measured output voltages of the PN‐TEGs with currents for three different distances between the electrodes, denoted as L, with values of 18, 10, and 2 mm (c.f. Figure 5c). The results revealed insights into the behavior of type‐V p–n junction TEGs. As the Δ*T* increases, the transverse output voltage and power output of both PN‐TEGs increase (c.f. **Figure** **4**). This phenomenon is attributed to the rise in thermal voltage with Δ*T* increasing from 1 mV for Δ*T* = 10 K to 7 mV for Δ*T* = 25 K. Furthermore, we found a remarkable enhancement in power output with a reduction in the distance between the electrodes *L* for a specific Δ*T*.

It is found that the open‐circuit voltage (*V*_OC_) of the printed PN‐TEGs does not change significantly with dimension while their internal resistances are altered. Therefore, the resultant power output increases with decreasing length L. This observation signifies the importance of electrode proximity, indicating that closer electrode configurations and higher thicknesses led to more efficient conversion of thermal energy into electrical power in these architectures. Interestingly, our study highlights the influence of thickness on the performance of the PN‐TEG as well for the same reason. The thicker PN‐TEG 80, with an 80 μm thickness, exhibits a lower internal resistance *R* across all distances the *L* and Δ*T* when compared to the thinner PN‐TEG 60. Consequently, PN‐TEG 80 consistently outperforms PN‐TEG 60 in terms of power output at all temperature differences. A maximum power output $P_{\text{max}}$ of 0.53 μW is achieved for Δ*T* = 10 K in the printed PN‐TEG 80.

## 3 Device Simulation

COMSOL Multiphysics version 6.1 package was utilized for 3D analysis and numerical investigation of the reported PN‐TEG (c.f. **Figure** **5**). From the numerical study, we can calculate the electric potential, power output, and temperature distribution within each module. The TE parametric simulation encompassed specific conditions: boundary condition (*q*_lateral_ = 0; no lateral heat flow), hot side (*T*_H_), and cold side (*T*_C_) temperatures set at 318 and 293 K, respectively. In the electrical analysis, a 0 V voltage was applied to one of the electrodes. In the thermal analysis, the *T* = *T*_H_ was assigned to the upper edge as a hot side while *T* = *T*_C_ was assigned to the lower edge as a cold side. To make the simulation more realistic, the experimental values of temperature‐dependent *σ*, *S*, and *κ* are imported into COMSOL. Figure 3 shows the measured TE materials parameters employed in the simulation study. The scheme of the printed PN‐TEG is shown in Figure 5a. The geometrical dimensions of the design are the thickness (*t*), width (*w*), and electrode spacing (*L*). The finite‐element method calculated the temperature profiles and *V*_OC_ for the suggested PN‐TEG via COMSOL Multiphysics. Figure 5b presents the temperature profile between the hot and cold surfaces and the corresponding *V*_OC_ is shown in Figure 5c.

**Figure 6:**
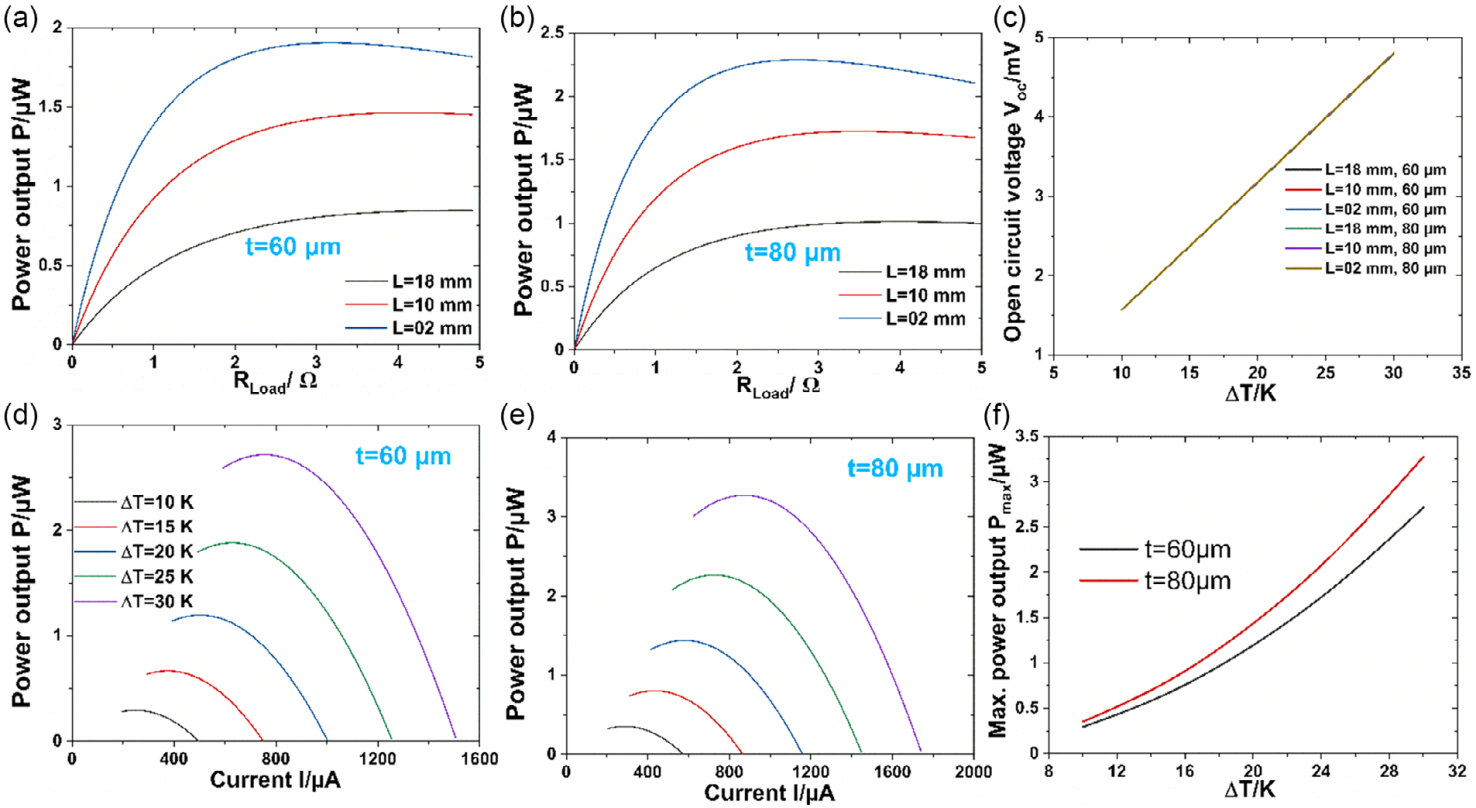
The output power versus the load resistance for the thickness of a) 60 μm, b) 80 μm, and c) the Δ*T*‐dependent open‐circuit voltage of both TEGs. d,e) The output power versus current of PN‐TEGs of *t* = 60 μm and *t* = 80 μm with a fixed *L* of 2 mm for different Δ*T*s between hot and cold surfaces. f) The variation of maximum power output with Δ*T*s for *t* = 60 and 80 μm.

The *L* and *w* do not affect the output voltage significantly. However, it influences the internal resistance (R) of the device and, consequently, the output power. Therefore, optimization of the *L* is crucial for increasing the output power. **Figure** **6**a,b shows the variation of output power with the load resistance for different *L* of the two PN‐TEGs with thicknesses of 60 and 80 μm. In this study, the temperature difference (Δ*Τ*) between the hot and cold sides is kept constant at 25 K. It can be noticed from these figures that the two PN‐TEGs show the same trend: the power increases with decreasing *L*. The maximum output powers of 1.87 and 2.26 μW are obtained for a device length of 2 mm for the two studied thicknesses 60 and 80 μm. The PN‐TEG thickness is one of the critical parameters that affect the PN‐TEG output power. The internal resistance R of the PN‐TEG decreases with increasing thickness, which enhances the power output. Therefore, it is found that the PN‐TEG with a thickness of 80 μm has a higher output power for all dimensions and Δ*Τ*s. Therefore, the following study is dedicated to investigating the impact of the TEG thickness on the output power. Figure 6d,e compares the output power for the introduced p–n junction TEG with thicknesses of 60 and 80 μm. Figure 6f illustrates the output power as a function of Δ*T* for the two devices with 60 and 80 μm thicknesses, respectively. In this study, the *T*_c_ is kept constant at 293 K, while *T*_H_ varies from 293 to 373 K with a step of 10 K. The numerical results demonstrate that the TEG output power increases with the temperature difference between the two surfaces for both 60 and 80 μm thicknesses.

## 4 Discussion

**Figure 7:**
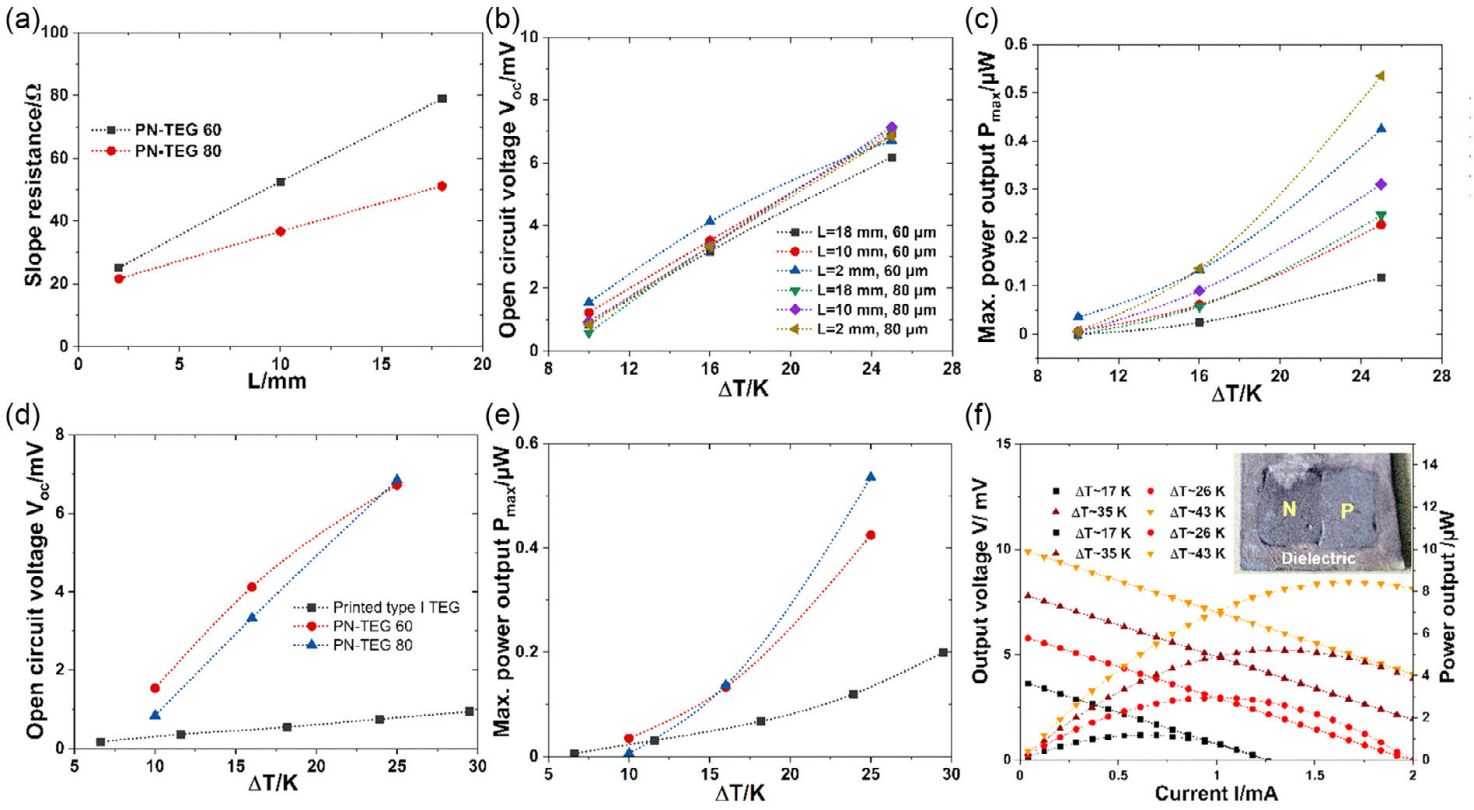
a) The dependence of averaged resistance on *L*. The variation of b) open‐circuit voltage and c) maximum power output with Δ*T* for different *L*. Comparison of d) the Δ*T*‐dependent open‐circuit voltage and e) maximum power output between printed conventional type‐I TEG and the printed PN‐TEG. f) The current‐dependent performance of the PN‐TEG with a thickness of 310 μm prepared by drop‐casting method.

The most interesting characteristic of the PN‐TEGs is their high transverse *V*_OC_ that remains unaffected by the change in device dimensions. Whereas the device's internal resistance R can be reduced considerably by modifying the device dimension. In this study, the output power of PN‐TEGs is found to be enhanced with decreasing L and increasing thickness signifying the reduction of device internal resistance R. Therefore, the average slope of the current–voltage (I–V) curve decreases with increasing L and thickness whereas the transverse open‐circuit output voltage remains unaffected and increases with increasing Δ*T* enhancing overall power output (c.f. **Figure** **7**a–c). The simulation results of the PN‐TEGs also predict similar trends.

Furthermore, the average thermal voltage per degree Kelvin (device Seebeck coefficient) of PN‐TEGs is found to be ≈190 μV K^−1^ while the simulated value amounts to ≈160 μV K^−1^. Whereas the device Seebeck coefficient for conventional printed type‐I TEG with the same width and thickness is only ≈29 μV K^−1^, one order of magnitude lower than the material Seebeck coefficient $\left(S_{p})-(-S_{n}\right)$. The I–V characteristic curve for printed type‐I TEG is given in Supporting Information (c.f. Figure S2, Supporting Information). The comparison of Δ*T*‐dependent *V*_OC_ between the PN‐TEGs and printed type‐I TEG is shown in Figure 7d. This difference can partly be attributed to the reduced thermal contact resistance in PN‐TEGs due to the absence of printed layers of electrodes and diffusion barriers. As the power output $P\propto V_{\text{OC}}^{2}/R$, despite exhibiting significantly lower internal resistance *R*, the printed type‐I TEG yields significantly lower *P* of ≈0.15 μW compared to ≈0.53 μW for the printed PN‐TEG in their respective maximum power points. Hence, a 14 times higher power output density of 5.3 μW cm^−2^ compared to “π‐type” printed TEGs is exhibited by the PN‐TEG. To check the stability, characterization of the PN‐TEG is repeated after around 6 months of the device fabrication, and power output is found to be similar (c.f. Figure S3, Supporting Information). The power outputs of the PN‐TEGs, however, are found to be lower compared to the simulated results. The device simulation predicts a maximum power output for PN‐TEG 80 with *L* = 2 mm of 1.5 μW. The discrepancy between experimental and simulated results can be explained by the different internal resistances. The average open‐circuit thermovoltage of the printed PN‐TEG is ≈18% higher than the simulated value, primarily attributable to the prevailing imperfect p–n junction with higher interfacial resistance. Ideally, if there is no physical direct contact between p‐ and n‐type legs (type I), the charges flow through only the electrode. Hence, the majority of carriers in both p‐ and n‐type legs move vertically from the hot to the cold side giving rise to approximately equal (if $\left|S_{n}\right|\approx \left|S_{p}\right|$) and opposite voltage at the cold side. Hence, the *V*_OC_ should be ≈ [$\left(S_{p})-(-S_{n}\right)$] × Δ*T* on the cold side. However, for a perfect PN‐TEG, the majority of charge carriers diffuse across the interface creating a vortex‐like current path creating an electrostatic potential approximately half of its type I counterpart observed by simulation. The higher *V*_OC_ of the printed PN‐TEG indicates that less charge carrier diffusion occurs across the interface due to its higher resistance, which is more than one order of magnitude higher than the simulated value. Therefore, most of the majority carriers accumulated on the cold side giving rise to higher electrostatic potential. Hence, the higher effective internal resistance due to the formation of a p–n junction is mainly responsible for the lower power output. To check the effect of overlap area on the device performance, two printed PN‐TEGs 80 with overlap widths of ≈0.2 and ≈10 mm, respectively, for an electrode distance of 15 mm are characterized (c.f. Figure S4, Supporting Information). The maximum power output of the PN‐TEG with an overlap of ≈0.2 mm is found to be 0.87 μW, which is slightly higher than 0.78 μW for the overlap of ≈10 mm. The output voltage of both PN‐TEGs is similar; however, the higher resistance for the PN‐TEG with an overlap of ≈10 mm is responsible for the lower power output. To realize the potential application of the PN‐TEG, a thicker and wider PN‐TEG with a thickness of ≈310 μm and a width of 8 mm has been fabricated using the drop‐casting method. The PN‐TEG yields almost six times higher power output of ≈3 μW than the PN‐TEG 80 for Δ*T* = 27 K as the junction area increases proportionally. The highest power out of 8.3 μW is exhibited for Δ*T* = 43 K. The results indicate that a single PN‐TEG can potentially yield tens of microwatts which is sufficient for IoT applications.

## 5 Conclusion

High‐performance printed TE materials for fabricating printed TEGs do not guarantee the proportionate conversion efficiency of the TEGs. High electrical and thermal contact resistance results in a low current and thermal voltage of a conventional printed TEG with “π‐type” geometry, thus causing low power output. Here, we report printed p–n junction type (type V) PN‐TEGs using the Bi–Sb–Te‐based p‐ and n‐type printed films minimizing the effect of thermal contact resistances. The PN‐TEGs display a promising approach to improving conversion efficiency in printed TEGs. Both experimental and simulation findings highlight the substantial influence of PN‐TEGs’ dimensions on their power outputs. The power output increases as the area decreases and the thickness of the printed TE legs increases. This is because of the reduction of the device's internal resistance, and the contrasting adverse effects observed in conventional “π‐type” printed TEGs. Additionally, we fabricated a conventional “π‐type” printed TEG and studied its performance. The optimized PN‐TEGs with a single thermocouple deliver ≈2.5 times higher power output compared to the “π‐type” printed TEGs.

## 6 Experimental Section

6.1

6.1.1

##### Materials

The following materials were used in the experiment: ingots of n‐type Bi_2_Te_2.7_Se_0.3_ and p‐type Bi_0.5_Sb_1.5_Te_3_ (EVERREDtronics), copper powder (spheroidal) (10–25 μm, 98%, Sigma‐Aldrich), Se powder (100 mesh, ≥99.5% trace metals basis, Sigma Aldrich), polyvinylpyrrolidone (PVP) (average Mw ≈40 000, Sigma Aldrich), N‐methyl‐2‐pyrrolidone (NMP) (anhydrous, 99.5%, Sigma‐Aldrich), and anodized aluminum substrate.

##### Preparation of Printable Inks and p–n Junction

The n‐type and p‐type TE inks were synthesized using Bi_2_Te_2.7_Se_0.3_ (n‐BT) and Bi_0.5_Sb_1.5_Te_3_ (p‐BST) TE particles respectively, following a previously established procedure. Ground p‐BST and n‐BT TE powders were mixed with an IB containing Cu, Se, and PVP in NMP solvent. The resulting mixtures underwent ball milling in a Fritsch Planetary Mill PULVERISETTE 5 premium line at 200 rpm for 45 min. The printable ink compositions contained 5 wt% of Cu–Se metal powder for p‐BST and 10 wt% of that for n‐BT. These inks were then printed onto flexible anodized aluminum substrates using a semi‐automated ROKUPRINT screen‐printing machine. The p‐type and n‐type films were printed adjacently with a slight overlap forming a p–n junction using two screens (600 × 300 90–40 year 22° Hitex). Following printing, the p–n junction was dried at 343 K for 5–10 min and subsequently underwent sintering using an oven with N_2_ environment at 623 K for 1 h.

##### Characterization for the Printed Materials and the p–n Junction TE Element

The temperature‐dependent electrical conductivity (*σ*) and Seebeck coefficient (*α*) of the individual printed n‐ and p‐type TE materials were determined using a Linseis HCS 10 instrument. The associated relative errors for *α* and *σ* measurements were 10% and 6%, respectively. Microstructural analyses were conducted with an FEI Quanta 650 environmental SEM in backscattered electron modes equipped with a solid‐state detector and a Schottky field emitter operated with 5 and 15 kV. The performance of the p–n junction TE elements was analyzed using a maximum power point tracking method by a KEITHLEY Source Measuring Unit 2601. The detailed device characterization setup was described in a previous report.^[^ ^25^ ^]^

## Conflict of Interest

The authors declare no conflict of interest.

## Supporting information

Supplementary Material

## Data Availability

The data that support the findings of this study are available from the corresponding author upon reasonable request.
