# Supplementary material for: Printed Lateral p–n Junction for Thermoelectric Generation
Source: Small Sci. 2024 Aug 13;4(11):2400257. doi: 10.1002/smsc.202400257 (PMC11934974; doi:10.1002/smsc.202400257)
Supplement: Supplementary file 1 — Supplementary Material [file SMSC-4-2400257-s001.pdf]

# Supporting Information

*Md Mofasser Mallick*, Leonard Franke, Mohamed Hussein, Andres Georg Rösch, Zhongmin Long , Yolita M. Eggeler, and Uli Lemmer**

M.-M. Mallick*, L. Franke, M. Hussein, A.-G. Rösch, U. Lemmer*

Light Technology Institute, Karlsruhe Institute of Technology (KIT), 76131 Karlsruhe, Germany.

Email: uli.lemmer@kit.edu; mofasser.mallick@kit.edu.

M. Hussein, U. Lemmer

Institute of Microstructure Technology, Karlsruhe Institute of Technology (KIT), 76344 Eggenstein-Leopoldshafen, Germany

M. Hussein

Department of Physics, Faculty of Science, Ain Shams University, 11566, Cairo, Egypt

Z. Long, Y.-M. Eggeler

Laboratory for Electron Microscopy, Karlsruhe Institute of Technology (KIT), 76131 Karlsruhe, Germany.

**Supporting information S-1:** Measurement setup

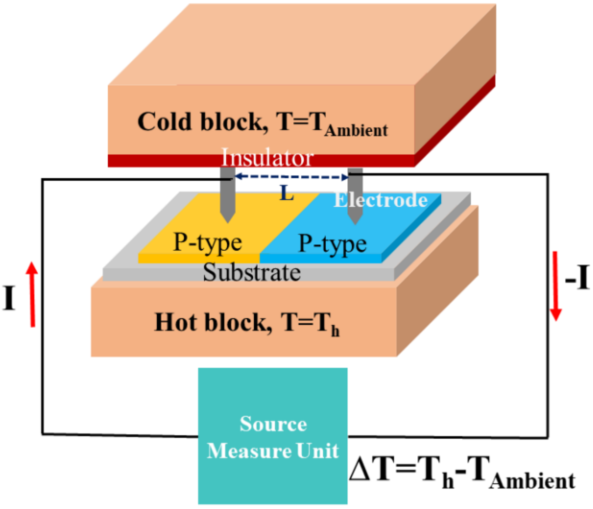


**Figure S 1:** Schematic diagram of the PN TEG measuring setup using maximum power point tracking method.

**Supporting information S-2:** Performance of printed conventional type-I TEG

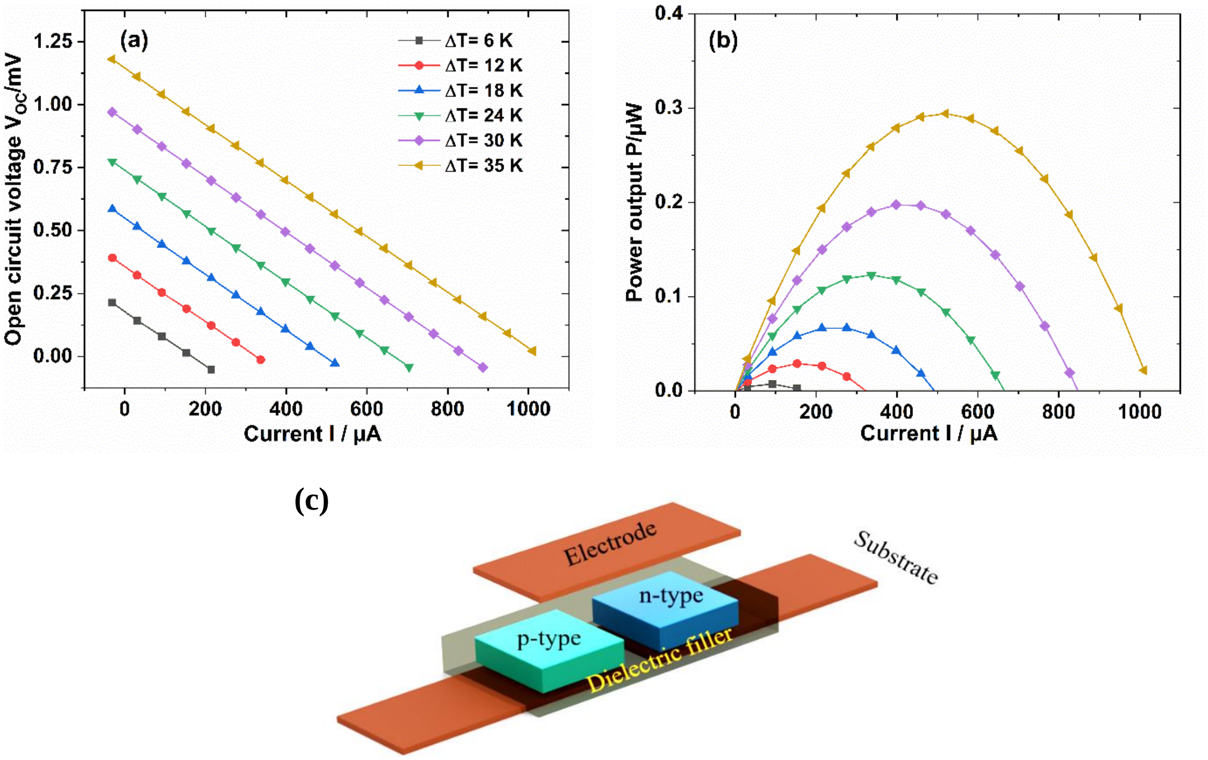


**Figure S2:** The variation of the (a) open circuit voltage ($V_{OC}$) and (b) power output with current of the printed conventional type-I TEG. (c) The device structure of the printed type-I TEG.

**Supporting information S-3:** Stability of the PN-TEG

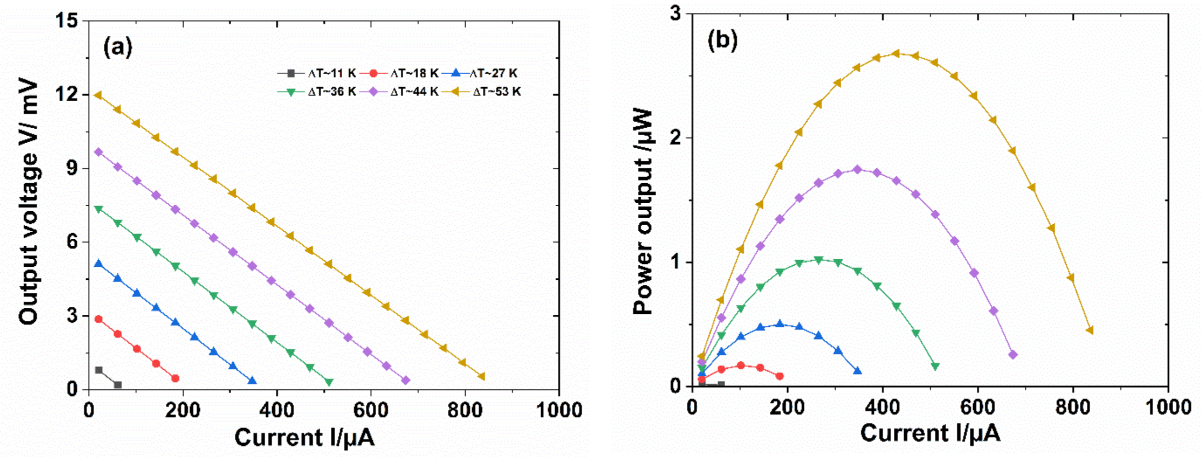


**Figure S3:** The variation of the (a) open circuit voltage ($V_{OC}$) and (b) power output with current of the printed PN-TEG with a thickness of 80 µm. The measurement is repeated after around 6 months of the device fabrication. The power output is found to be ~0.5 µW for a *ΔT=27 K* which is close to the first measured value of 0.53 µW.

**Supporting information S-4:** Effect of overlap area on PN-TEG performance

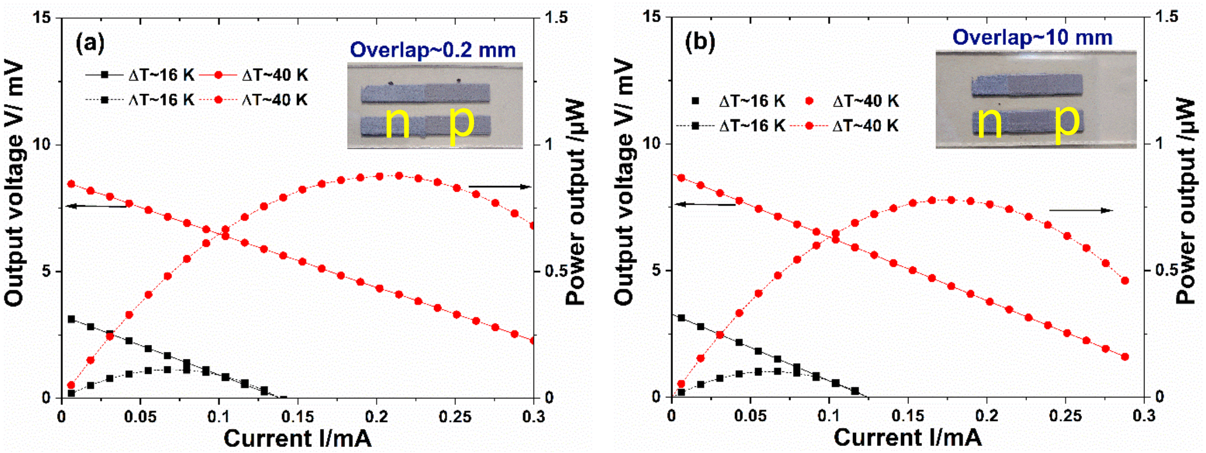


**Figure S4:** The performance of a printed PN-TEG 80 with an overlap width of ~0.2 mm (a) and ~10 mm (b) for an electrode distance of 15 mm. The maximum power output of the PN-

TEG with an overlap of ~0.2 mm is found to be 0.87 μW, which is slightly higher than 0.78 μW for the overlap ~10 mm due to higher interface resistance.
